# Population Genomics Reveals Panmixia in Pacific Sardine (*Sardinops sagax*) of the North Pacific

**DOI:** 10.1111/eva.70154

**Published:** 2025-09-04

**Authors:** Gary C. Longo, Katie D′Amelio, Wes Larson, Concepción Enciso Enciso, Jorge Torre, Jeremiah J. Minich, Todd P. Michael, Matthew T. Craig

**Affiliations:** ^1^ Ocean Associates, Inc. Under Contract to the National Oceanic and Atmospheric Administration, National Marine Fisheries Service Southwest Fisheries Science Center La Jolla California USA; ^2^ National Oceanographic and Atmospheric Administration, National Marine Fisheries Service Alaska Fisheries Science Center, Auke Bay Laboratories Juneau Alaska USA; ^3^ Centro Regional de Investigación Acuícola y Pesquera de Ensenada Instituto Mexicano de Investigación en Pesca y Acuacultura Sustentables, Carretera Tijuana‐Ensenada Km 97.5 Parque Industrial Fondeport Ensenada México; ^4^ Comunidad y Biodiversidad, A.C Isla del Peruano 215, col. Lomas de Miramar Guaymas México; ^5^ The Plant Molecular and Cellular Biology Laboratory The Salk Institute for Biological Studies California USA; ^6^ National Oceanographic and Atmospheric Administration, National Marine Fisheries Service Southwest Fisheries Science Center La Jolla California USA

**Keywords:** Alosidae, California current, Clupeiformes, fisheries genomics, population structure

## Abstract

The spatial structure and dynamics of populations are important considerations when defining management units in organisms that are harvested as natural resources. In the Eastern Pacific, Pacific Sardine range from Chile to Alaska, the northernmost state of the United States (U.S.), and once supported an expansive and productive fishery. Along its North American range, it is hypothesized to comprise three subpopulations: a northern and southern subpopulation, which primarily occur off the coast of the U.S. and Baja California, Mexico (M.X.), respectively, and a third in the Gulf of California, M.X. We used low coverage whole genome sequencing to generate genotype likelihoods for millions of SNPs in 317 individuals collected from the Gulf of California, M.X., to Oregon, U.S., to assess population structure in Pacific Sardine. Differentiation across the genome was driven by variation at several putative chromosomal inversions ranging in size from ~21 MB to 0.89 MB, although none of the putative inversions showed any evidence of geographic differentiation. Our results support panmixia across an impressive ~4000 km range.

## 1 Introduction

The spatial structure of populations is an important consideration when defining management units (i.e., “stocks”) in fishes that are harvested as natural resources. Accurate estimates of migration rates and delineation of population boundaries facilitate the construction of spatially appropriate management units that ensure that demographically distinct population segments are managed appropriately. In addition, if multiple populations of a target species are harvested by or monitored in a single fishery, it is desirable to estimate the contribution of each distinct population (Christensen et al. 2022). In doing so, an accurate alignment of population structure and management units (i.e., coherent dimensionality) can be achieved while simultaneously ensuring the use of the best quality data in stock assessments (Andersson et al. 2024; Berger et al. 2021; Cadrin 2020; Cadrin and Secor 2009; Cadrin et al. 2023). Because population structure is unknown for many fish species, management units are frequently defined based on factors such as geopolitical or jurisdictional boundaries, despite the fact that this may violate the unit stock assumptions of many stock assessment models (Cadrin et al. 2023).

In North America, the Pacific Sardine ( *Sardinops sagax* ) historically supported fisheries in Canada, the United States, and Mexico, and at one point represented the largest fishery in the Western Hemisphere (Norton and Mason 2005). Following rapid declines in biomass leading to the decimation of the fishery in the mid‐1940s, and a contraction of Pacific Sardine's geographic distribution to its core area off Baja California, a resurgence of Pacific Sardine into the northern portion of its range during the late 1980s and early 1990s prompted a flurry of scientific and management interest in potential population structure. If population structure existed, managers were interested in knowing if the northern areas were being repopulated by individuals with different genetic lineages or life history traits that could impact productivity (Hedgecock 1984; MacCall 1984). Previous hypotheses had been posed (e.g., Marr and Murphy 1960) that three subpopulations of Pacific Sardine existed along its North American range (see Craig et al. 2025 for a review of this topic). While the geographic limits of these subpopulations are not consistently defined in the literature, their ranges are generally as follows: the northern subpopulation (NSP) ranges from Alaska, U.S., to Northern Baja California, M.X., the southern subpopulation (SSP) ranges from Central California, U.S., to the southern tip of Baja California, M.X., and the Gulf of California subpopulation (GOCSP) ranges from the southern tip of the Pacific coast of Baja California, M.X., to the Gulf of California (Kuriyama et al. 2024; Zwolinski and Demer 2023; Félix‐Uraga et al. 2005; Yau 2022; Figure S1). The three subpopulations are thought to have synchronous, seasonal migrations that result in overlap of their absolute geographic ranges (i.e., the NSP and SSP have range overlap, and SSP and GOCSP have range overlap), but they are not thought to occupy the same space at the same time (Zwolinski and Demer 2023; Félix‐Uraga et al. 2005).

While early studies using serological antigen response purported to show population structure in Pacific Sardine (Sprague and Vrooman 1962; Vrooman 1964), this antiquated method has been shown to be incapable of doing so (see Craig et al. 2025, for a review of this topic). More recently, studies using genetic techniques have supported panmixia in Pacific Sardine (Adams and Craig 2024; Bowen and Grant 1997; Grant et al. 1998; Gutiérrez Flores 2007; Hedgecock et al. 1989; Lecomte et al. 2004), which is not surprising for a highly mobile marine species with a ~45‐day pelagic larval duration (Ahlstrom 1954) and large effective population size. While these studies supported panmixia, they were based on mitochondrial haplotypes or a small number of nuclear markers and thus may not have been able to detect subtle population structure that would be relevant to management. Recent advances in genomic tools have drastically increased marker resolution and provided vital information relevant to fisheries management by allowing for the detection of weak differentiation as well as adaptive differentiation and genomic structural variation (Bernatchez et al. 2017; Andersson et al. 2024). For example, Enbody et al. (2021) used a genomics approach and showed that localized, ecological adaptation in European eels ( *Anguilla anguilla* ) is a result of phenotypic plasticity in a species that lacks geographic genetic differentiation. In the European Sardine ( *Sardina pilchardus* ), Da Fonseca et al. (2024) employed a population genomics approach that confirmed previously identified genetic differences and detected outlier loci related to otolith formation, which have been used to distinguish populations (Jemaa et al. 2015). Adaptive genetic variation associated with genomic structural variation (e.g., chromosomal inversions) has been identified in diverse fishery species such as Atlantic Cod ( *Gadus morhua* ; Barth et al. 2019; Berg et al. 2016), Atlantic Herring ( *Clupea harengus* ; Han et al. 2020), and Lingcod ( *Ophiodon elongatus* ; Longo et al. 2020), among others.

Pacific Sardine management in the U.S. is a complicated endeavor given the estimated geographic ranges of the hypothesized NSP and SSP, which not only overlap with each other but also span the international boundary between the United States and Mexico. Because a majority of the estimated geographic range of the NSP lies within U.S. territorial waters, only the NSP is managed by the U.S., while the SSP and GOCSP are managed by Mexico. In the absence of any morphological differences, allocation of fishery landings or scientific survey biomass estimates to the NSP or SSP is difficult. To accomplish this, a potential habitat model was created for the NSP (Demer and Zwolinski 2014; Zwolinski et al. 2011; Zwolinski and Demer 2023). This model was developed using satellite‐derived sea surface temperature, sea surface height, chlorophyll *a* concentration, and the distribution of sardine eggs to predict probable habitat for the NSP of Pacific Sardine (Demer and Zwolinski 2014; Zwolinski et al. 2011; Zwolinski and Demer 2023). Individuals are assigned to the NSP if the environmental conditions fit within a defined envelope (see Zwolinski and Demer 2023 for details). All others in U.S. waters are assumed to be part of the SSP. Due to low estimated biomass levels of the NSP in the U.S., the directed fishery has been closed since 2015, with exceptions for the small‐volume live‐bait fishery and research activities.

Given that the current management scheme for Pacific Sardine in the U.S. is based on the supposition that population structure is present, and that genetic methods have failed to detect such structure, there exists both a need and an opportunity to use genomic methods to provide higher resolution genetic data that may help to resolve this conflict. Given the use of environmental data in assigning fish to the NSP or SSP, the opportunity also exists to associate potential genetic differentiation with environmental variables. Herein, we apply low coverage, whole genome sequencing (lcWGS) to assess population structure in Pacific Sardine. In using this approach, we interrogated the entire genome of 317 Pacific Sardine collected from Oregon, U.S., to the Gulf of California, M.X., and show that Pacific Sardine represent a single, genetically well‐mixed population that spans an impressive ~4000 km of coastline. We also show that the management of Pacific Sardine suffers from incoherent dimensionality.

## 2 Methods

### 2.1 Sample Collection

Most samples used in analyses here were previously sequenced for a lcWGS study reporting the presence of Japanese Sardine (*Sardinops melanosticta*) in the Eastern Pacific (see Longo et al. 2024; BioProject PRJNA1094947). Briefly, these samples were either collected during the 2021 and 2022 California Current Ecosystem Surveys (CCES) conducted by the Southwest Fisheries Science Center (SWFSC) from Tillamook, Oregon, U.S., to Ensenada, Baja California, M.X., obtained from a chartered fishing vessel in Long Beach, California, U.S., in 2022, or collected from Magdalena Bay, Baja California Sur, M.X., in 2022. Twenty‐three additional samples were sequenced for this study, which were collected in 2023 from the Gulf of California, M.X. (GOC).

### 2.2 Library Preparation and Low Coverage Whole Genome Sequencing

Sequence data for the GOC samples were generated on a different sequencing run than the previously reported samples (Longo et al. 2024). As such, there is a possibility that batch effects (i.e., differences attributed to library preparation and/or sequencing) may bias the sequence data (Lou and Therkildsen 2022). To test for a batch effect, we included 8 previously sequenced samples (Longo et al. 2024) in the sequencing run with the 23 individuals from the GOC (see supplemental information for details).

For the newly sequenced individuals, genomic DNA was extracted from muscle tissue stored in 100% ethanol using Qiagen DNAeasy Blood & Tissue 96 extraction kits (Qiagen Inc., Valencia, CA) following the manufacturer's protocol. Extractions were run on a standard 2% agarose gel to screen for high molecular weight DNA and were then quantified using a PicoGreen fluorescence on a BioTek Synergy HTX microplate reader; only samples with > 5 ng/μL were selected. After 10 ng of DNA from each high‐quality extraction was plated, the 96‐well plate was sealed with a microporous sealing film and stored at room temperature until liquid evaporated from all wells. DNA was then fragmented and tagged with a universal Nextera overhang following the Nextera DNA Library Prep Kit protocol (Illumina Inc., San Diego, CA) with some modifications (i.e., using 1/20th of recommended reagents). Tagmented libraries were then amplified with low‐cycle PCR and barcoded using Illumina Nextera dual‐indices at concentrations of 5 μM. Additional amplification and the attachment of Illumina P5 and P7 sequencing primers was carried out using another round of low‐cycle PCR. Tagmented and indexed samples were then normalized (≦ 25 ng) using 96‐well SequelPrep Normalization Plates following the manufacturer's protocol and then pooled for each plate. Pooled libraries were cleaned using AMPure XP beads (Beckman Coulter Inc., Brea, CA) and eluted in 20 μL of TLE buffer. Final lcWGS sequencing libraries were then visualized on an E‐Gel (ThermoFisher Inc., Waltham, MA) to determine whether the ideal size range (200–1000 bp) was achieved and quantified using a Qubit 2.0 dsDNA HS Assay (ThermoFisher Inc., Waltham, MA). Two lcWGS libraries, each containing 96 individuals, were sequenced on a single lane with S4 chemistry (2 × 150 bp paired end) on an Illumina NovaSeq 6000 at the Azenta facility (Burlington, MA).

### 2.3 lcWGS Data Filtering and Analyses

We generally followed Laura Timm's lcWGS analysis pipeline (see https://github.com/letimm/WGSfqs‐to‐genolikelihoods for scripts). For lcWGS analyses, haplotype 1 (hap 1) of the Pacific Sardine reference genome (Longo et al. 2024; BioProject PRJNA1094947) was indexed using BWA v0.7.17 (Li and Durbin 2009) and Samtools v1.11 *faidx* (Li et al. 2009) after excluding contigs that were not incorporated into putative chromosomes. Raw lcWGS data were de‐multiplexed into forward and reverse fastq files for each individual. We used FastQC v0.11.9 (Andrews 2010) and MultiQC v1.14 (Ewels et al. 2016) to check the sequence quality of individual raw reads. We trimmed adapters and polyG tails from raw fastq files using Trimmomatic v0.39 (Bolger et al. 2014) and fastp v0.23.2 (Chen et al. 2018), respectively, and again assessed the sequence quality on trimmed reads using FastQC and MultiQC. Next, we aligned trimmed reads to the reference genome using BWA. Samtools was then used to clean up read pairings and flags from BWA with *fixmate*, convert sam to bam files, filter non‐unique and poor‐quality mappings before sorting read pairs by mapping coordinate. After bam files were built, duplicate reads were detected and removed with Picard *MarkDuplicates* v2.23.9 (http://broadinstitute.github.io/picard/) and overlapping paired‐end reads were clipped with bamtools *clipOverlap* v2.5.1 (Barnett et al. 2011) to generate final bam files. We then used Samtools *depth* to tally alignment depth in all individuals. Individuals with < 1× mean depth of coverage were filtered from downstream analyses. To reduce potential sequencing depth bias, we performed targeted down‐sampling. Target down‐sampling depths were drawn from the distribution of mean individual depths calculated from the data.

### 2.4 lcWGS Genotype Likelihood Calls and Analyses

Preliminary analyses were performed to test for batch effects among sequencing runs and library preparations, which we did not detect (see supplemental information for details, Figure S2). BAM files from 295 previously analyzed samples and the 22 GOC samples passing quality filters here were used to calculate genotype likelihoods (GLs) for all sites using ANGSD v0.933 (Korneliussen et al. 2014). Low‐quality base calls and mapped reads were excluded with minimum quality and mapping quality set to 15 (−minQ 15 and ‐minMapQ 15). We set the minimum depth to the total number of individuals (‐setminDepth 317) and the maximum depth to the total number of individuals multiplied by 20 (‐setmaxDepth 6340), which should exclude mtDNA but still retain regions sequenced at high coverage. We set the threshold for minor allele frequency to 5% (−minMaf 0.05) and the *p*‐value filter for polymorphic sites to 10^−8^ (‐SNP_pval 1e‐10).

To explore potential genetic structure in our data, we conducted principal component analysis (PCA) using PCAngsd (Meisner and Albrechtsen 2018) based on SNPs from the full genome as well as for each chromosome independently. The covariance matrices were then imported into R (R Core Team 2024) to perform eigen decomposition and visualization. We also estimated individual admixture proportions with NGSadmix (Skotte et al. 2013) testing *K* values from 1 to 10 with 3 iterations. The Evanno method (Evanno et al. 2005) and likelihood scores were used to identify the most likely *K* value (number of genetic clusters). Initial PCAs suggested that putative chromosomal structural variation was driving observed patterns in the whole genome PCA. To look for potential population structure outside of structural variation, we excluded chromosomes that appeared to harbor chromosomal inversions and then reran PCA and admixture analyses (testing *K* values 1–6 with 3 iterations).

We also estimated population‐level *F*_ST_ using GLs between sampling locations with ≥ 14 individuals passing QF as well as based on subpopulation assignments to the NSP from the sardine potential habitat model that were graciously provided by Juan Zwolinski at the NOAA Fisheries SWFSC. Samples along the Pacific coast of the U.S. and Baja California that were not assigned to the NSP were assumed to be a part of the SSP. Samples from Magdalena Bay, Baja California Sur were collected in July; thus, following Félix‐Uraga et al. (2005) they were assumed to be part of the hypothesized GOCSP. In order to determine weighted pairwise *F*_ST_ among groups, site allele frequency likelihoods were calculated in ANGSD using the same filtering criteria as above. Global and genome‐wide *F*_ST_ were calculated among groups using the folded site frequency spectrum (‐realSFS). To assess the significance of global *F*_ST_, we tested if the observed *F*_ST_ value fell significantly outside a distribution from permuting individuals, assuming *F*_ST_ values follow an exponential distribution (Elhaik 2012). For comparisons between subpopulations, we generated Manhattan plots to visualize genetic differentiation across the genome. We tested for isolation by distance (IBD) among sampling sites by estimating the correlation coefficient between pairwise *F*_ST_ values and least‐cost path distances calculated in marmap (Pante and Simon‐Bouhet 2013) using a Mantel test (Mantel 1967) with 10,000 permutations in the r package vegan (Oksanen et al. 2024).

To better assess the size and patterns of divergence of chromosomal inversions, we computed locus‐specific *F*_ST_ values based on likely karyotype groups observed in chromosome‐specific PCAs for chromosomes 1, 2, 9, 11, 15, 18, and 20, and then generated Manhattan plots. Notably, these are putative inversions based on characteristic patterns observed in PCA, admixture analyses, and Manhattan plots. Confirmation of chromosomal inversions requires direct observation through methods such as cytogenetic analysis or direct sequencing of break points, which is beyond the scope of this study. Some analyses and most plotting were conducted in R (R core team 2024) with the use of several tidyverse packages (Wickham et al. 2019).

## 3 Results

### 3.1 Filtering, Depth of Coverage, Number of Individuals and Loci

**FIGURE 1:**
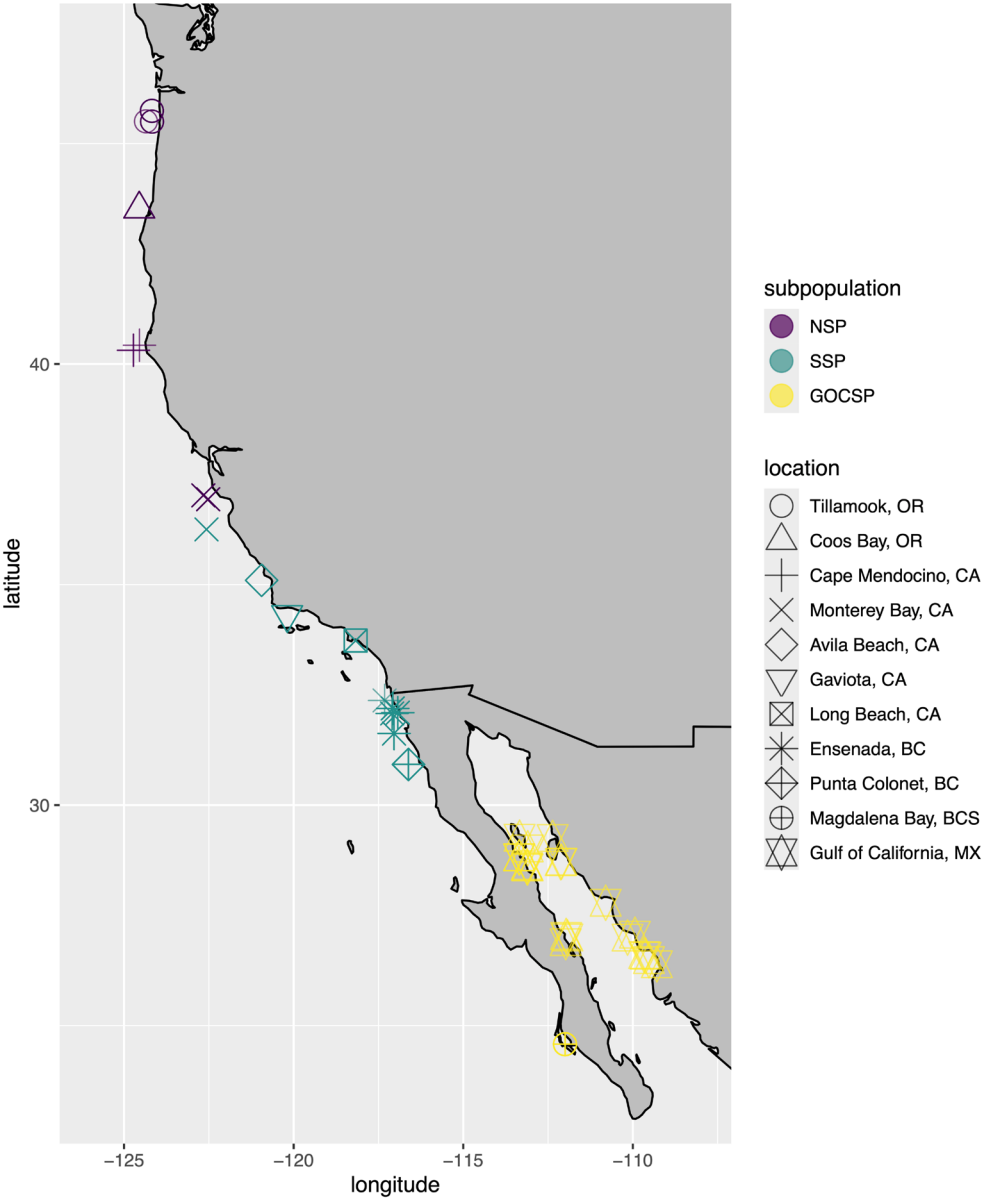
Sampling locations of 317 Pacific Sardine samples passing quality filters (BC, Baja California; BCS, Baja California Sur; CA, California; MX, Mexico; OR, Oregon). Colors correspond to subpopulation assignments (GOCSP, Gulf of California subpopulation; NSP, northern subpopulation; SSP, southern subpopulation).

After quality filtering, 317 Pacific Sardine individuals remained (295 previously sequenced samples and 22 of 23 newly sequenced GOC samples; Figure 1, Table 1) with a mean coverage of 2.97 (range 1.02–7.85). After targeted downsampling, mean coverage was 2.29 (range 1.02–4.39). SNP filtering parameters resulted in 9,819,187 polymorphic loci. Assignment of individuals to subpopulations resulted in 63 individuals assigned to the NSP, 183 to the SSP, and 71 to the GOCSP. When seven chromosomes with putative inversions were removed, 6,905,971 polymorphic sites remained.

**TABLE 1:** Number of individuals passing quality filter by sampling site.

Location	*n*
Tillamook, OR	14
Coos Bay, OR	15
Cape Mendocino, CA	3
Monterey Bay, CA	38
Avila Beach, CA	10
Gaviota, CA	29
Long Beach, CA	49
Ensenada, BC	44
Punta Colonet, BC	44
Magdalena Bay, BCS	49
Gulf of California, MX	22

Abbreviations: BC, Baja California; BCS, Baja California Sur; CA, California; MX, Mexico; OR, Oregon.

### 3.2 PCAs

**FIGURE 2:**
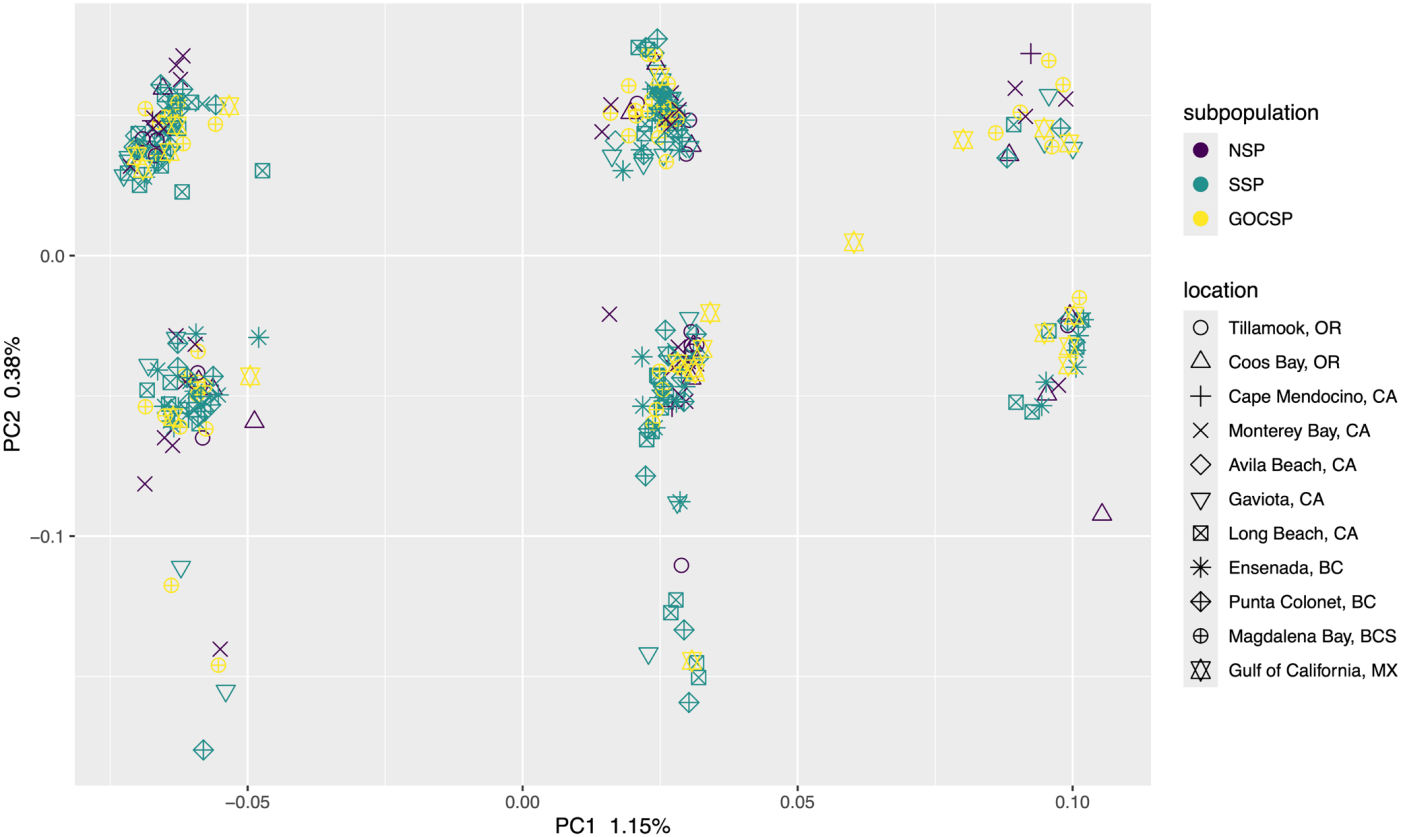
Principal component analysis on 9,819,187 polymorphic sites from 317 Pacific Sardine samples collected from Oregon, U.S., to the Gulf of California, M.X. Colors correspond to subpopulation assignments (GOCSP, Gulf of California subpopulation; NSP, northern subpopulation; SSP, southern subpopulation).

**FIGURE 3:**
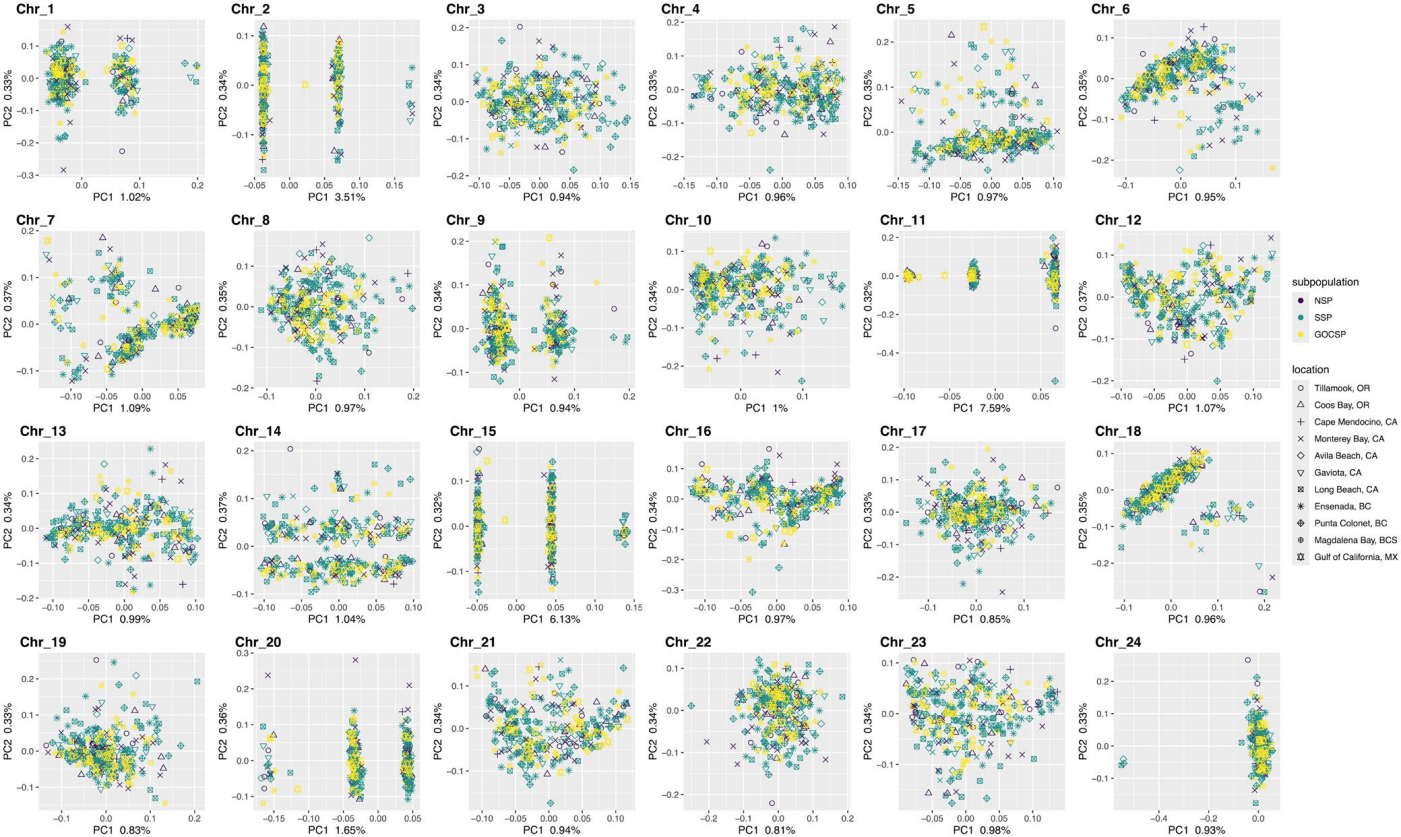
Chromosome‐specific principal component analyses for 317 Pacific Sardine samples collected from Oregon, U.S., to the Gulf of California, M.X. Colors correspond to subpopulation assignments (GOCSP, Gulf of California subpopulation; NSP, northern subpopulation; SSP, southern subpopulation).

PC1 explained 1.15% of the variation in the genome‐wide PCA and separated Pacific Sardine into three distinct groups, while PC2 explained 0.38% of the variation and also separated individuals into three groups, although clustering was less distinct below −0.1 (Figure 2). The PCA groups showed no apparent association with geographic sampling sites or subpopulation assignment. Chromosome‐specific PCAs showed a wide range of clustering patterns from definitively separated groups to no apparent pattern (Figure 3). Chromosomes 11, 15, and 2 exhibited the clearest differentiation along PC1, which explained 7.59%, 6.13%, and 3.51% of the variation, respectively. Notably, the separation observed on PC1 and PC2 in the genome‐wide PCA (Figure 2) is completely explained by PC1 scores from chromosomes 11 and 15, respectively (Figure 3; Figure S3). A single individual from the Gulf of California fell out between clear groups both in the whole genome PCA and on chromosome‐specific PCAs and was excluded from downstream population‐level analyses.

**FIGURE 4:**
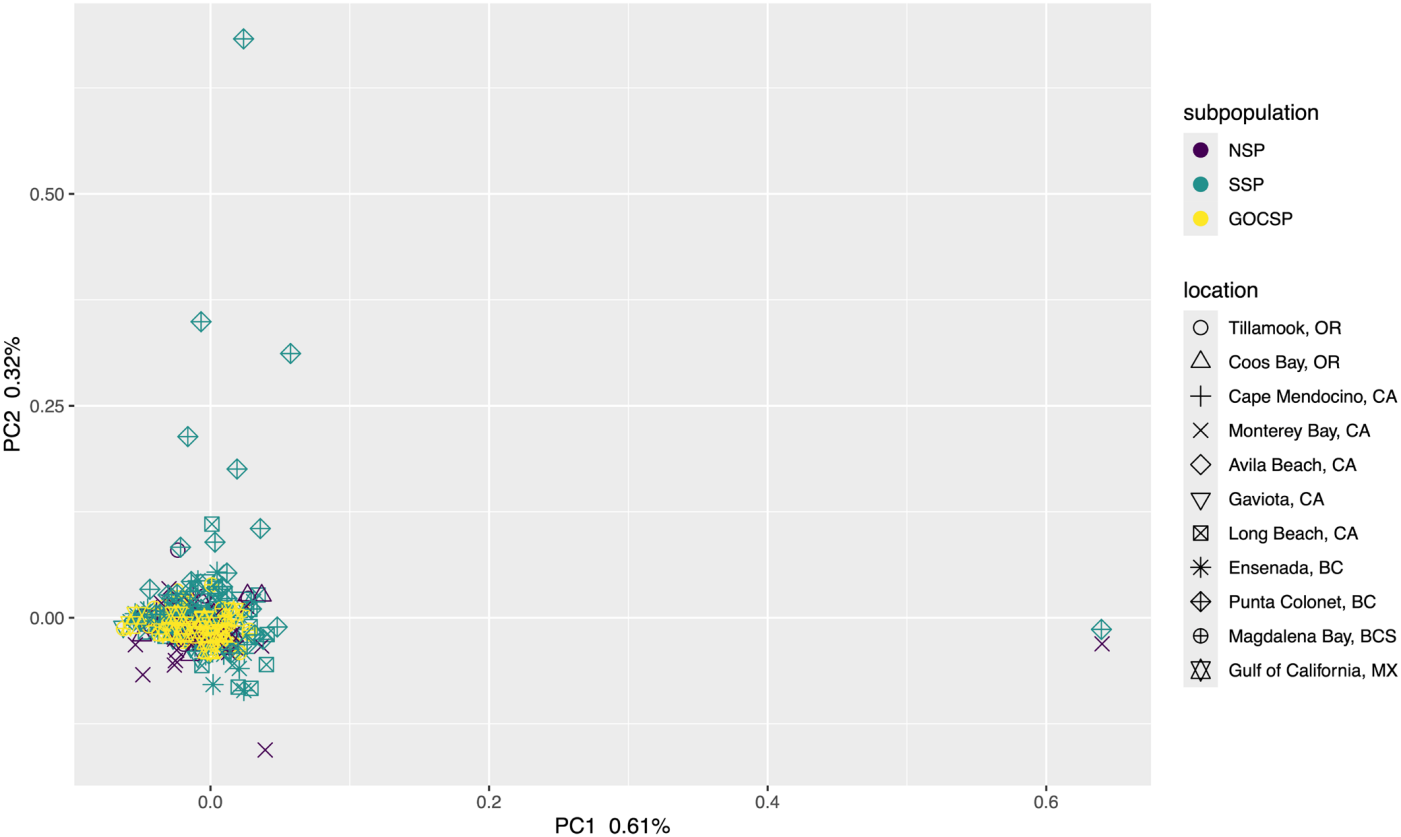
Principal component analysis excluding chromosomes with putative inversions (1, 2, 9, 11, 15, 18, & 20) on 6,905,971 polymorphic sites from 317 Pacific Sardine samples collected from Oregon, U.S., to the Gulf of California, M.X. Colors correspond to subpopulation assignments (GOCSP, Gulf of California subpopulation; NSP, northern subpopulation; SSP, southern subpopulation).

After chromosomes with putative inversions (see below for details) were removed, no genetic differentiation was detected among samples, which nearly all grouped together (Figure 4).

### 3.3 NGSadmix Analyses

**FIGURE 5:**
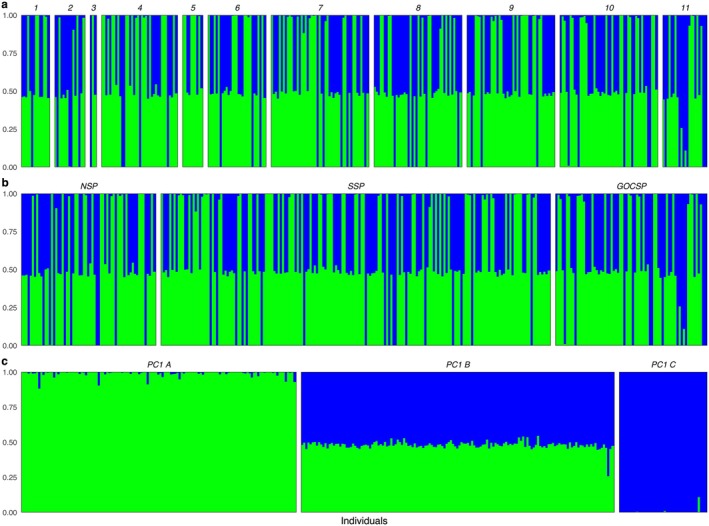
NGSadmix results for *K* = 2 on 9,819,187 polymorphic sites from 317 Pacific Sardine samples collected from Oregon, U.S., to the Gulf of California, M.X. Individuals are arranged based on (a) sampling sites latitudinally (1 = Tillamook, OR, 2 = Coos Bay, OR, 3 = Cape Mendocino, CA, 4 = Monterey Bay, CA, 5 = Avila, CA, 6 = Gaviota, CA, 7 = Long Beach, CA, 8 = Ensenada, BC, 9 = Punta Colonet, BC, 10 = Magdalena Bay, BCS, 11 = Gulf of California, M.X.), (b) putative subpopulation (GOCSP, Gulf of California subpopulation; NSP, northern subpopulation; SSP, southern subpopulation), and (c) groups separated by PC1 scores from the genome‐wide principal component analysis (PC1 A < 0, PC1 B > 0 & < 0.07, PC1 C > 0.07).

Admixture results for *K* = 2, which was the best supported *K* value by three orders of magnitude for the full data set (Table S1), assigned individuals almost entirely to one of the two genetic clusters (≥ 0.8) or nearly evenly to both (~0.5) in most cases (Figure 5). Frequency of assignments did not appear correlated with sampling locations or putative subpopulation identifications but correlated with PC 1 groupings from the genome‐wide PCA, which is identical to Chromosome 11–specific PC 1 groups (Figure 3; Figure S3).

When putative inversions were removed (see below for details), *K* = 2 again was identified as the most likely number of clusters, although support was much lower compared with the full data set (Table S2). However, NGSadmix failed to converge on individual assignment proportions across iterations, which is indicative of a lack of structure in the data (Anders Albrechsten, personal communication), results were not plotted.

### 3.4 *F*st and Isolation by Distance

**FIGURE 6:**
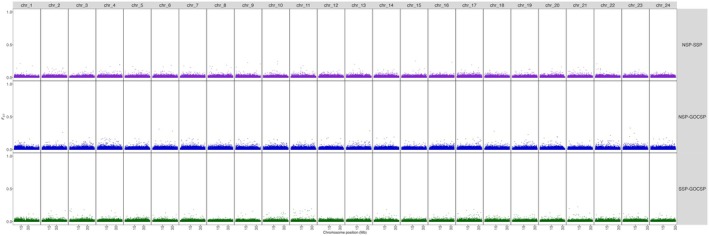
Manhattan plot aligning lcWGS polymorphic sites to the Pacific Sardine genome with locus‐specific *F*_ST_ based on pairwise comparisons between putative subpopulations (GOCSP, Gulf of California subpopulation; NSP, northern subpopulation; SSP, southern subpopulation).

The global weighted *F*_ST_ between putative subpopulations of Pacific Sardine ranged from 0.002 to 0.003 with no significant comparisons (Table 2). Manhattan plots of locus‐specific pairwise comparisons of putative subpopulations did not show any areas of elevated differentiation across the genome (Figure 6). Comparisons of *F*_ST_ between sampling sites ranged from 0.002 (Tillamook, Oregon vs. Coos Bay, Oregon) to 0.008 (Coos Bay, Oregon vs. Punta Colonet, Baja California) again with no significant comparisons (Table 3). The Mantel test found a nonsignificant correlation coefficient (*r* = 0.363, *p*‐value = 0.05072) between pairwise sampling site *F*_ST_ values and least‐cost path distances, suggesting no pattern of IBD. Notably, none of the pairwise *F*_ST_ values used in the IBD analysis were significant.

**TABLE 2:** Pairwise *F*_ST_ comparisons between subpopulations estimated with the full dataset and corresponding *p*‐values.

Comparison	*F*_ST_	*p*
NSP—SSP	0.002	0.956
NSP—GOCSP	0.003	0.977
SSP—GOCSP	0.002	0.964

Abbreviations: GOCSP, Gulf of California subpopulation; NSP, northern subpopulation; SSP, southern subpopulation.

**TABLE 3:** Pairwise *F*_ST_ comparisons based on the full data set between sampling sites with ≥ 14 individuals are in the lower diagonal with *p*‐values in the upper diagonal. Tillamook, OR (1TI), Coos Bay, OR (2CB), Monterey Bay, CA (3MO), Gaviota, CA (4GA), Long Beach, CA (5LB), Ensenada, Baja California (6EN), Punta Colonet, Baja California (7PC), Magdalena Bay, Baja California Sur (8MA), and Gulf of California, Mexico (9GC).

	1TI	2CB	3MO	4GA	5LB	6EN	7PC	8MA	9GC
1TI		1	0.939	1	1	0.997	0.967	0.995	1
2CB	0.0026		0.845	0.978	0.941	0.949	0.883	0.923	1
3MO	0.0080	0.0082		0.951	0.914	0.881	0.913	0.939	0.863
4GA	0.0058	0.0062	0.0046		0.961	0.948	0.933	0.976	1
5LB	0.0083	0.0083	0.0040	0.0045		0.93	0.919	0.953	0.951
6EN	0.0082	0.0080	0.0043	0.0046	0.0037		0.891	0.902	1
7PC	0.0083	0.0085	0.0042	0.0046	0.0038	0.0040		0.905	0.897
8MA	0.0085	0.0084	0.0039	0.0044	0.0034	0.0038	0.0038		0.878
9GC	0.0043	0.0046	0.0061	0.0049	0.0059	0.0055	0.0062	0.0064	

### 3.5 Putative Inversions

Manhattan plots of comparisons between putative inversion karyotypes on chromosomes 1, 2, 9, 11, 15, and 20 showed elevated *F*_ST_ blocks ranging from 0.89 MB on chromosome 9–21.79 MB on chromosome 11 (Figure S4). The percent of variance explained by PC1 in each chromosome‐specific PCA (Figure 3) correlated with putative inversion size.

## 4 Discussion

Here, we used lcWGS to assess the population structure of Pacific Sardine from the coast of Oregon, U.S., to the Gulf of California, M.X., and found strong genetic evidence for panmixia. We also detected high levels of structural variation in the Pacific Sardine genome, with several chromosomes characterized by putative inversions. However, none of the structural variants shows any correlation with geographic sampling sites or purported subpopulations. These structural variants could potentially be associated with phenotypic variability that is not correlated with environmental variables, such as color patterns or behavior (see Wellenreuther and Bernatchez 2018 for a review), or may be non‐adaptive.

The geographic range of the Pacific Sardine in the Northeast Pacific spans at least 38° latitudinal degrees and encompasses a diverse set of environmental conditions, particularly as related to temperature. This environmental heterogeneity has the potential to drive local adaptation and/or reduce geneflow, resulting in environmentally driven population structure, which could be detected through analyses such as genotype–environment associations (GEA; Grummer et al. 2019). Indeed, advances in analytical methods such as the use of redundancy analysis have allowed for even subtle GEA to be revealed (Forester et al. 2018); however, many of these analytical tools are currently not built under a probabilistic framework, which is used in GL‐based methods. However, because assignment to the NSP in Pacific Sardine is accomplished through the use of an environmentally derived potential habitat model, the subpopulation comparisons in our analyses can approximate a more robust analysis of GEA. Our results show that despite the heterogenous nature of Pacific Sardine habitats, environmental factors do not appear to be driving selection or population structure. This is consistent with the ability and propensity of Pacific Sardine to perform long‐distance, annual migrations (Hart 1944; Clark and Jansen 1945; Craig et al. 2025) that span large portions of this diverse set of conditions, as well as their temporally protracted and geographically extensive spawning habits (see Craig et al. 2025, for a review of this topic).

Although our results confirm panmixia, we detected relatively high amounts of genomic structural variation in Pacific Sardine. Specifically, PCAs for chromosomes 1, 2, 9, 11, 15, 18, and 20 exhibit a pattern associated with chromosomal inversions where homokaryotypes for inverted and uninverted karyotypes group separately, with heterokaryotypes (i.e., individuals heterozygous for inverted and uninverted regions) falling out between (Wellenreuther and Bernatchez 2018). NGSadmix results for the full data set and Manhattan plots for chromosome‐specific PC1 groupings, which correspond to the putative inversion karyotypes, also display patterns consistent with chromosomal inversions (Wellenreuther and Bernatchez 2018). Structural variants (e.g., chromosomal inversions) can allow for differentiation in the face of gene flow (Nosil et al. 2009); however, none of these putative structural variants appear to be correlated with putative subpopulations or sampling sites. Inversion karyotypes that carry adaptive phenotypes associated with environmental variables generally exhibit geographically structured patterns (Wellenreuther and Bernatchez 2018), such as latitudinal clines (Longo et al. 2020; Campbell and Hale 2024), or show clear geographic distributions (Berg et al. 2016; Barth et al. 2019; Han et al. 2020). There are cases of inversion karyotypes with no clear geographic structuring in other marine fishes, such as Sablefish ( *Anoplopoma fimbria* ; Timm et al. 2024) and Atlantic Halibut ( *Hippoglossus hippoglossus* ; Kess et al. 2021). Some of the putative inversions detected in Pacific Sardine did not appear to be present at a frequency high enough to detect in their sibling species, Japanese Sardine (*Sardinops melanosticta*; Longo et al. 2024), which share a relatively recent common ancestor (Bowen and Grant 1997), indicating that these structural rearrangements likely evolved recently. Alternatively, some structural rearrangements could have arisen before speciation but subsequently drifted to fixation in one taxon. Further work is warranted to better understand the underlying genes and potential adaptive nature of the putative chromosomal inversions characterized here.

Except for the fact that Pacific Sardine are managed in the U.S. under the hypothesis of population structure, our genetic results supporting panmixia are not unexpected. Pacific Sardine are iteroparous spawners and, while temporal and geographical peaks in spawning activity occur, have a protracted spawning season and broad spawning habitat (see Craig et al. 2025, for a review of this topic). Eggs hatch at around 2.5 days (Garrison and Miller 1982; Matarese et al. 1989) and pelagic larval duration is roughly 45 days (Ahlstrom 1954). As adults, Pacific Sardine are capable of rapid, long‐distance seasonal movements from central Baja California, M.X., to the state of Washington, U.S. (Clark and Jansen 1945; Clark and Marr 1955). In addition, even at low biomass levels, Pacific Sardine exist in vast numbers, thus effective population sizes are high and genetic drift is therefore low (Waples 2025; Wright 1931). All of these factors contribute to gene flow that is sufficient to reduce the likelihood of genetic population structure developing in the absence of strong selection or effective dispersal barriers.

Many studies over the past few decades have pointed to the spawning habits of the NSP and SSP of Pacific Sardine as a differentiating characteristic (but see references and review in Craig et al. 2025, for why this is not well supported). Some studies have gone so far as to characterize spawning in the NSP and SSP as being spatiotemporally segregated (e.g., Demer and Zwolinski 2014; Zwolinski and Demer 2023). While often not explicitly mentioned, there is an implication that this segregated spawning results in some degree of reproductive isolation which could factor into the maintenance of the hypothesized subpopulation structure. However, our genomic results corroborate previous genetic findings suggesting panmixia as even sardines from the Gulf of California, M.X., are undifferentiated from those in the Pacific northwest of the U.S.

Although we detect no signs of genetic isolation, we cannot completely rule out some degree of demographic isolation, which could be obscured by large effective population sizes and low genetic drift (Waples et al. 2008). However, intraspecific genetic differentiation has been observed in other coastal pelagic clupeiform species characterized by large effective population sizes but with clearly distinct spawning habitats or timing (Han et al. 2020; Petrou et al. 2021; Teske et al. 2021). A notable example is the closely related congener found off South Africa, which is currently valid as a distinct species, *Sardinops ocellatus* , although often referred to as *S. sagax* in the literature due to previous taxonomic uncertainty. For most of the year, these sardines exhibit a discontinuous distribution with centers of biomass separated by the boundary between the Atlantic and Indian Oceans near Cape Agulhas (Coetzee et al. 2008; Grantham et al. 2011). These groups have distinct spawning temperatures and different nursery habitats (McGrath et al. 2020; Mhlongo et al. 2015; Miller et al. 2006). One of these groups exhibits migratory behavior to reach spatially discrete and temporally discontinuous upwelling regions to which it is adapted. Using genome‐scale data, Teske et al. (2021) demonstrated that these groups represent genetically differentiated populations. If Pacific Sardine exhibited a similar reproductive pattern in which spatiotemporally segregated spawning took place (e.g., Demer and Zwolinski 2014; Zwolinski and Demer 2023), it is reasonable to expect that similar selective/adaptive genetic signals would be present that we did not detect with our genome‐scale data. Similarly, Félix‐Uraga et al. (2004) suggested that adult Pacific Sardine from the NSP and SSP are adapted to specific temperature profiles. Again, no such signals of adaptive differentiation were present in our data that would support such a scenario.

The lcWGS data analyzed here support panmixia in Pacific Sardine from the Pacific Northwest, U.S., to the Gulf of California, M.X., which is generally consistent with previous genetic studies. Our results do not provide support for the current management framework of Pacific Sardine in the U.S. and suggest that multiple management units have been defined for a single biological population. While there is more risk in managing discrete management units as a single population as opposed to managing a panmictic population as distinct populations, neither are ideal (Berger et al. 2021; Cadrin 2020; Cadrin et al. 2023; Kerr et al. 2017; Laikre et al. 2005). Such misalignment of management and biological units, or incoherent dimensionality *sensu* Berger et al. (2021), should be avoided if possible. Although splitting of a single biological population into multiple management units may be convenient in some cases due to jurisdictional or political considerations (e.g., management of the Sablefish; Kapur et al. 2024), this can affect not only the biological response to harvest, but also management assessments and regulatory responses to them. That is, assessments may produce biased management metrics (e.g., reference points), especially if the management unit is not scaled to account for the entire life history of the biological population (e.g., spawning, recruitment, movements) in both time and space. This is because population processes are effectively averaged across the management area. This bias can be inflated by demographic leakage between management units, for example, if there is movement between them that is unaccounted for (Berger et al. 2021). As an example of this in Pacific Sardine, splitting of the biological population into a northern and southern subunit ignores the empirically derived evidence of their north/south movements, the length of which differs over the ontogeny of an individual and which may span nearly the entire range of both the NSP and SSP (Clark and Jansen 1945; Clark and Marr 1955; reviewed in Craig et al. 2025). Ultimately, the results of this study show that no genetic population structure exists in Pacific Sardine and, coupled with the lack of other data supporting population structure (Craig et al. 2025; Erisman et al. 2025), demonstrate that current management practices suffer from incoherent dimensionality.

**Table:** 

*K*	Delta *K*
1	Inf
2	985,781,998
3	719708.82
4	19870.09
5	23177.16
6	19284.22
7	16670.03
8	25762.3
9	37,573
10	17701.77

*K*	Delta *K*
1	Inf
2	243748.7
3	131401.04
4	85593.2
5	40486.94
6	57443.15

## Conflicts of Interest

The authors declare no conflicts of interest.

## Supporting information


**Figure S1:** Generalized distributions of the hypothesized northern subpopulation (blue), southern subpopulation (yellow), and Gulf of California subpopulation (orange) of Pacific Sardine. While these subpopulations are not thought to fully occupy the same region at the same time, their absolute geographic ranges are thought to overlap.

**Figure S2:** Principal component analysis testing for batch effect. Newly sequenced samples included 22 Gulf of California, M.X. individuals that passed quality filters, 8 previously sequenced individuals from Oregon, U.S. (to test for batch effect), and an individual previously identified as *Sardinops melanosticta* with a GTseq panel targeting mitochondrial DNA collected in 2014 (sample 735–18; see Longo et al. 2024). These were analyzed with all 345 samples passing quality filters from a prior *Sardinops* lcWGS analysis (see Longo et al. 2024 for details on prior analysis and GTseq panel). The right grouping (PC1 > 0.1; 50 individuals) represent Japanese Sardine ( *S. melanosticta* ) and the left grouping (PC1 < 0; 326 individuals) represent Pacific Sardine ( *S. sagax* ). Mitochondrial introgressed individuals (i.e., individuals with Pacific Sardine nuclear genomes and Japanese Sardine mitogenomes) are labeled (MTC071422_F08 and 735–18).

**Figure S3:** Principal component analysis (PCA) on 9,819,187 polymorphic sites from 317 Pacific Sardine samples collected from Oregon, U.S., to the Gulf of California, M.X., with individuals color‐coded based on (a) PC1 groupings from chromosome 11 PCA and (b) chromosome 15 PCA.

**Figure S4:** Putative chromosomal inversions visualized with Manhattan plots with locus‐specific *F*_ST_ based on pairwise comparisons between putative karyotypes.

**Table S1:** The Evanno method output (Δ*K*) for NGSadmix runs using the full data set testing *K* number of clusters with 3 replicates.

**Table S2:** The Evanno method output (Δ*K*) for NGSadmix runs using the data set excluding chromosomes with putative inversions testing *K* number of clusters with 3 replicates.

**Appendix S1:** eva70154‐sup‐0007‐AppendixS1.docx.

## Data Availability

Raw lcWGS data are deposited in the SRA NCBI sequence repository under the BioProject PRJNA1094947 will be made available upon acceptance.
