# Supplementary figures and images for: Population Genomics Reveals Panmixia in Pacific Sardine (Sardinops sagax) of the North Pacific
Source: Evol Appl. 2025 Sep 4;18(9):e70154. doi: 10.1111/eva.70154 (PMC12409727; doi:10.1111/eva.70154)

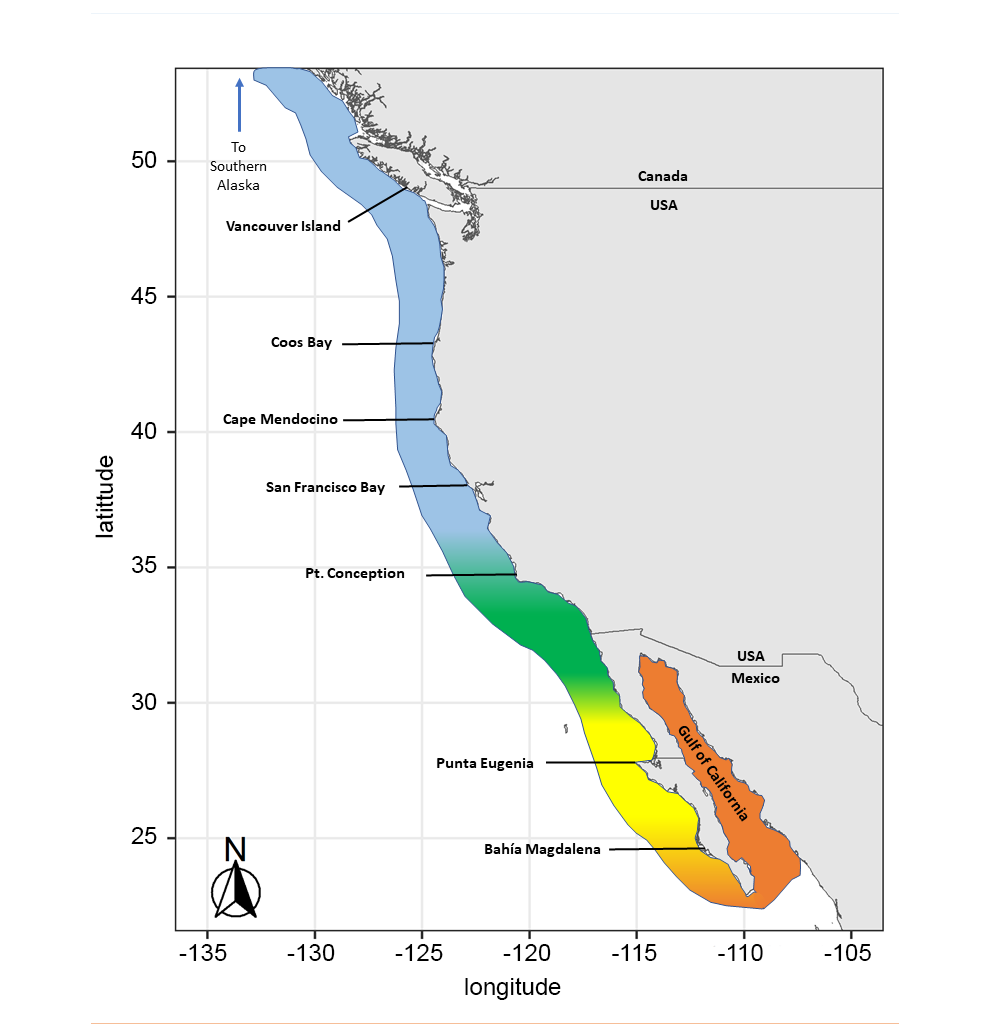

Supplement: Supplementary file 1 — Figure S1: Generalized distributions of the hypothesized northern subpopulation (blue), southern subpopulation (yellow), and Gulf of California subpopulation (orange) of Pacific Sardine. While these subpopulations are not thought to fully occupy the same region at the same time, their absolute geographic ranges are thought to overlap. [file EVA-18-e70154-s004.png]

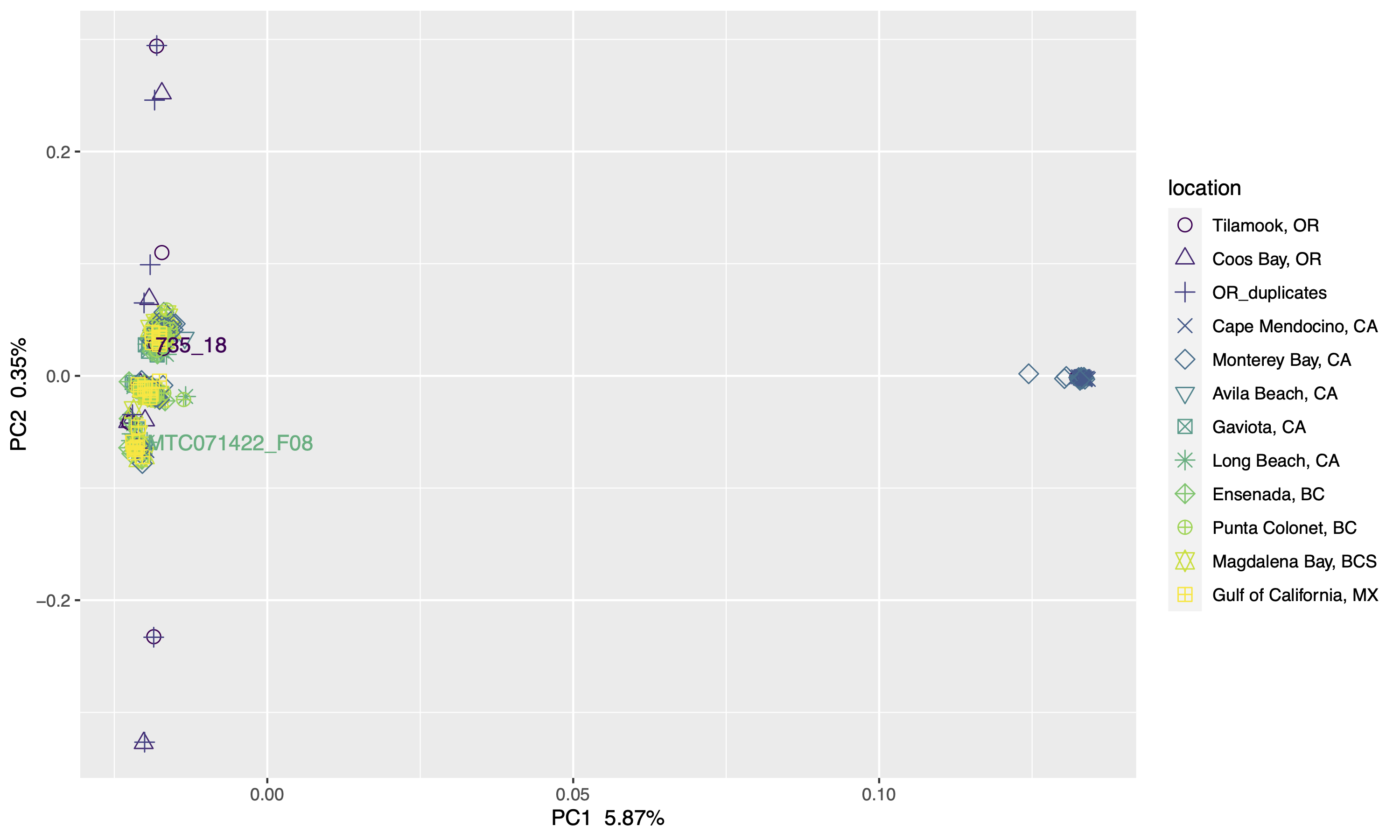

Supplement: Supplementary file 2 — Figure S2: Principal component analysis testing for batch effect. Newly sequenced samples included 22 Gulf of California, M.X. individuals that passed quality filters, 8 previously sequenced individuals from Oregon, U.S. (to test for batch effect), and an individual previously identified as Sardinops melanosticta with a GTseq panel targeting mitochondrial DNA collected in 2014 (sample 735–18; see Longo et al. 2024). These were analyzed with all 345 samples passing quality filters from a prior Sardinops lcWGS analysis (see Longo et al. 2024 for details on prior analysis and GTseq panel). The right grouping (PC1 > 0.1; 50 individuals) represent Japanese Sardine ( S. melanosticta ) and the left grouping (PC1 < 0; 326 individuals) represent Pacific Sardine ( S. sagax ). Mitochondrial introgressed individuals (i.e., individuals with Pacific Sardine nuclear genomes and Japanese Sardine mitogenomes) are labeled (MTC071422_F08 and 735–18). [file EVA-18-e70154-s007.png]

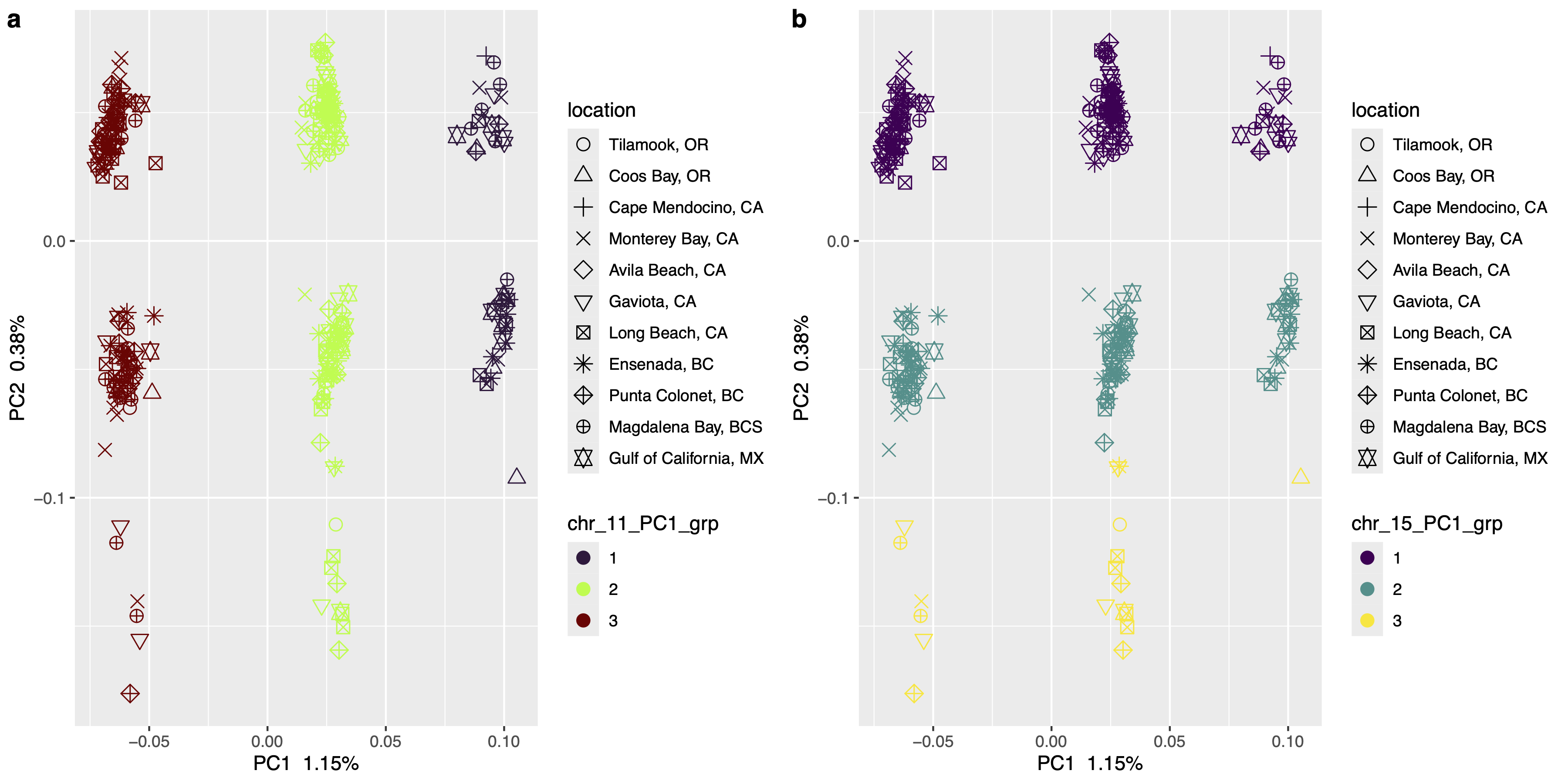

Supplement: Supplementary file 3 — Figure S3: Principal component analysis (PCA) on 9,819,187 polymorphic sites from 317 Pacific Sardine samples collected from Oregon, U.S., to the Gulf of California, M.X., with individuals color‐coded based on (a) PC1 groupings from chromosome 11 PCA and (b) chromosome 15 PCA. [file EVA-18-e70154-s002.png]

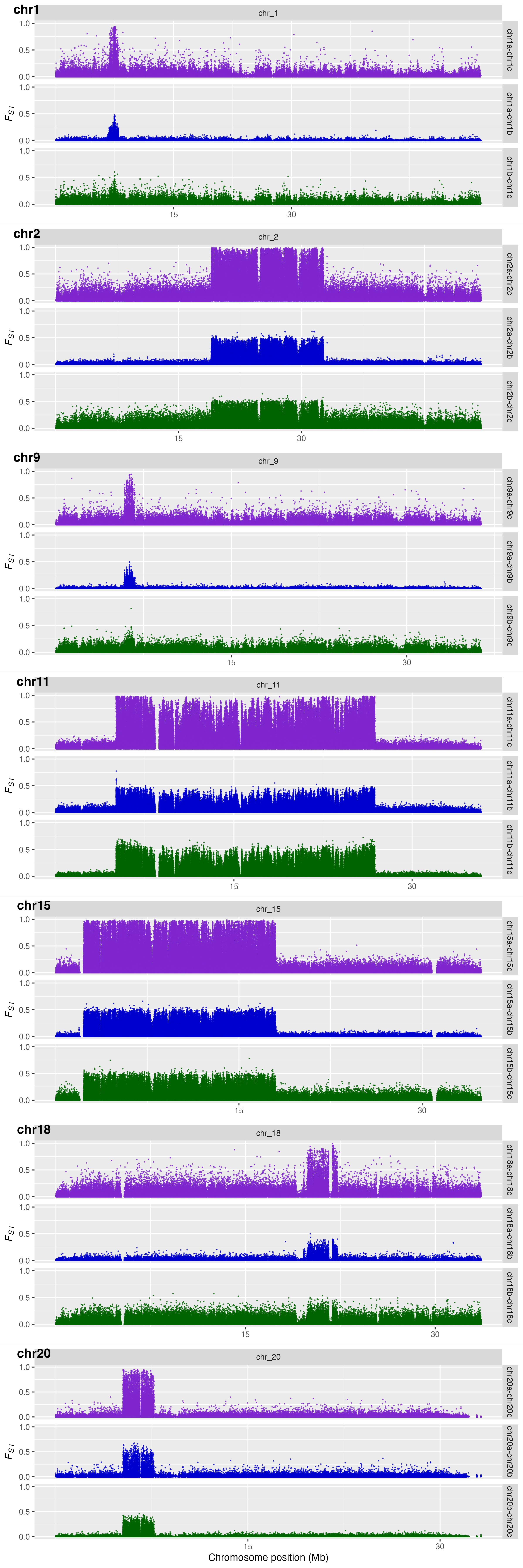

Supplement: Supplementary file 4 — Figure S4: Putative chromosomal inversions visualized with Manhattan plots with locus‐specific F ST based on pairwise comparisons between putative karyotypes. [file EVA-18-e70154-s003.jpg]
