# Supplementary material for: Population Genomics Reveals Panmixia in Pacific Sardine (Sardinops sagax) of the North Pacific
Source: Evol Appl. 2025 Sep 4;18(9):e70154. doi: 10.1111/eva.70154 (PMC12409727; doi:10.1111/eva.70154)
Supplement: Supplementary file 7 — Appendix S1: eva70154‐sup‐0007‐AppendixS1.docx. [file EVA-18-e70154-s001.docx]

**Populations Genomics Confirms Panmixia in Pacific Sardine (*Sardinops sagax*) of the North Pacific**

**Supplementary Information**

**Testing for batch effect**

*lcWGS genotype likelihood calls and PCA*

Samples sequenced for this study that passed quality filters, which included 8 previously sequenced individuals, 22 GOC samples, and an individual identified as *S. melanosticta* (with a GTseq panel targeting mitochondrial DNA) collected in 2014 on the CCES, were analyzed with all 345 samples passing quality filters from a prior *Sardinops* lcWGS analysis (see Longo et al. 2024 for details on prior analysis and GTseq panel).

BAM files from 345 previously analyzed samples (including 50 Japanese Sardine) and 31 samples passing QF sequenced in this study (22 GOC samples, 8 duplicate Oregon samples, and an individual identified as Japanese Sardine based on mitochondrial DNA ) were used to calculate genotype likelihoods for all sites using ANGSD v0.933 (Korneliussen et al., 2014). Low-quality base calls and mapped reads were excluded with minimum quality and mapping quality set to 15 (-minQ 15 and -minMapQ 15). We set the minimum depth to the total number of individuals (-setminDepth 376) and the maximum depth to the total number of individuals multiplied by 20 (-setmaxDepth 7520), which should exclude mtDNA but still retain regions sequenced at high coverage. We set the threshold for minor allele frequency to 5% (-minMaf 0.05) and the p-value filter for polymorphic sites to 10^-8^ (-SNP_pval 1e-10).

To check for batch effect, we ran a PCA with all samples to evaluate if the 8 duplicate samples sequenced in separate libraries plotted differently. All duplicates overlapped or fell out very close to each other indicating no batch effect (Fig. S2). Note PCA with duplicate samples can distort PC scores (hence the duplicate Oregon samples separating on PC2 from the other Pacific Sardine).
